# Cellular uptake, cytotoxicity and DNA-binding studies of the novel imidazoacridinone antineoplastic agent C1311

**DOI:** 10.1038/sj.bjc.6690702

**Published:** 1999-09

**Authors:** A M Burger, T C Jenkins, J A Double, M C Bibby

**Affiliations:** Tumor Biology Center at the University of Freiburg, Freiburg, D-79106, Germany; School of Chemical and Life Sciences, University of Greenwich, London, SE18 6PF, UK; Clinical Oncology Unit, University of Bradford, Bradford, BD7 1DP, UK

**Keywords:** C1311, HT29 cells, colorectal cancer, cytotoxicity, DNA intercalation, lysosomes

## Abstract

C1311 is a novel therapeutic agent with potent activity against experimental colorectal cancer that has been selected for entry into clinical trial. The compound has previously been shown to have DNA-binding properties and to inhibit the catalytic activity of topoisomerase II. In this study, cellular uptake and mechanisms by which C1311 interacts with DNA and exerts cytotoxic effects in intact colon carcinoma cells were investigated. The HT29 colon cancer cell line was chosen to follow cellular distribution of C1311 over a time course of 24 h at drug concentrations that just inhibited cell proliferation by 50% or 100%. Nuclear uptake of C1311 and co-localization with lysosomal or mitochondrial dyes was examined by fluorescence microscopy and effects on these cellular compartments were determined by measurement of acid phosphatase levels, rhodamine 123 release or DNA-binding behaviour. The strength and mode of DNA binding was established by thermal melting stabilization, direct titration and viscometric studies of host duplex length. The onset of apoptosis was followed using a TUNEL assay and DNA-fragmentation to determine a causal relationship of cell death. Growth inhibition of HT29 cells by C1311 was concomitant with rapid drug accumulation in nuclei and in this context we showed that the compound binds to duplex DNA by intercalation, with likely A/T sequence-preferential binding. Drug uptake was also seen in lysosomes, leading to lysosomal rupture and a marked increase of acid phosphatase activity 8 h after exposure to C1311 concentrations that effect total growth inhibition. Moreover, at these concentrations lysosomal swelling and breakdown preceded apoptosis, which was not evident up to 24 h after exposure to drug. Thus, the lysosomotropic effect of C1311 appears to be a novel feature of this anticancer agent. As it is unlikely that C1311-induced DNA damage alone would be sufficient for cytotoxic activity, lysosomal rupture may be a critical component for therapeutic efficacy. © 1999 Cancer Research Campaign


*British Journal of Cancer* (1999) **81**(2), 367–375
© 1999 Cancer Research Campaign
Article no. bjoc.1999.0702

### Cellular uptake, cytotoxicity and DNA-binding studies of the novel imidazoacridinone antineoplastic agent C1311

**AM Burger[1], TC Jenkins[2], JA Double[3] and MC Bibby[3]**

[1]Tumor Biology Center at the University of Freiburg, D-79106 Freiburg, Germany; [2]School of Chemical and Life Sciences, University of Greenwich, London SE18 6PF, UK; [3]Clinical Oncology Unit, University of Bradford, Bradford BD7 1DP, UK

**Summary** C1311 is a novel therapeutic agent with potent activity against experimental colorectal cancer that has been selected for entry into clinical trial. The compound has previously been shown to have DNA-binding properties and to inhibit the catalytic activity of topoisomerase II. In this study, cellular uptake and mechanisms by which C1311 interacts with DNA and exerts cytotoxic effects in intact colon carcinoma cells were investigated. The HT29 colon cancer cell line was chosen to follow cellular distribution of C1311 over a time course of 24 h at drug concentrations that just inhibited cell proliferation by 50% or 100%. Nuclear uptake of C1311 and co-localization with lysosomal or mitochondrial dyes was examined by fluorescence microscopy and effects on these cellular compartments were determined by measurement of acid phosphatase levels, rhodamine 123 release or DNA-binding behaviour. The strength and mode of DNA binding was established by thermal melting stabilization, direct titration and viscometric studies of host duplex length. The onset of apoptosis was followed using a TUNEL assay and DNA-fragmentation to determine a causal relationship of cell death. Growth inhibition of HT29 cells by C1311 was concomitant with rapid drug accumulation in nuclei and in this context we showed that the compound binds to duplex DNA by intercalation, with likely A/T sequence-preferential binding. Drug uptake was also seen in lysosomes, leading to lysosomal rupture and a marked increase of acid phosphatase activity 8 h after exposure to C1311 concentrations that effect total growth inhibition. Moreover, at these concentrations lysosomal swelling and breakdown preceded apoptosis, which was not evident up to 24 h after exposure to drug. Thus, the lysosomotropic effect of C1311 appears to be a novel feature of this anticancer agent. As it is unlikely that C1311-induced DNA damage alone would be sufficient for cytotoxic activity, lysosomal rupture may be a critical component for therapeutic efficacy.

© 1999 Cancer Research Campaign

**Keywords:** C1311; HT29 cells; colorectal cancer; cytotoxicity; DNA intercalation; lysosomes

Imidazoacridinones are a new class of potent antineoplastic agents which have been developed by Konopa and co-workers at the Technical University of Gdansk, Poland (Cholody et al, 1990, 1992). The design of these compounds was based on structure–activity relationship studies of the chemotherapeutic agent mitoxantrone. The diaminoalkyl groups ('daag') in the side-chains of mitoxantrone had previously been found to be a prerequisite for the biological activity of this drug (Konopa and Skladanowski, 1987; Cheng and Cheng, 1989). The attachment of daag to known DNA-intercalating acridinone moieties resulted in the C13xx imidazoacridinone series of compounds, which showed anti-tumour activity in vitro and in vivo in various tumour model systems (Kusnierczyk et al, 1994).

We have recently reported that the most potent imidazoacridinone analogue, C1311 (Figure 1), induces inhibition of experimental human and murine colorectal cancer cell growth. The human colon cancer xenograft HT29 showed marked tumour growth delay (100 mg $kg^{-1}$ = 211.4%, $P < 0.001$) after a single-dose intraperitoneal (i.p.) bolus injection (Burger et al, 1996). Based on these and other xenograft data (unpublished) obtained by European Organisation for Research and Treatment of Cancer and Screening and Pharmacology Group (EORTC-SPG) screening laboratories, C1311 was selected for further evaluation and will soon enter clinical phase I trials.

Several groups have been studying possible mechanisms underlying the antiproliferative activity of C1311 in vitro and most reports describe the cellular response as being associated with DNA-related events or damage, including DNA binding (Berger et al, 1996; Burger et al, 1996), topoisomerase II inhibition (Skladanowski et al, 1996) and G2 arrest (Augustin et al, 1996; Lamb and Wheatley, 1996). However, due to the fluorescent nature of imidazoacridinone derivatives, we and others followed the cellular distribution of these compounds and observed the rapid accumulation of C1311 fluorescence not only in nuclei, but also in other cytoplasmic organelles (Berger et al, 1996; Burger et al, 1996; Skladanowski et al, 1996).

The present study was designed to define the cytosolic compartment and the relevance of the involvement of these organelles in the anti-tumour efficacy of C1311. Cellular uptake and the mechanism by which C1311 interacts with DNA and exerts cytotoxic effects in intact colon carcinoma cells were investigated. We describe the cellular mechanisms of C1311 in relation to its structural features supporting that DNA intercalation and lysosomal rupture are the lethal lesions induced at drug concentrations causing total growth inhibition in HT29 colon cancer cells. Moreover, the lysosomotropic effect of C1311 might be a critical component for C1311 therapeutic efficacy against colorectal cancer in vitro and in vivo.

*Received 4 November 1998*
*Revised 18 Febuary 1999*
*Accepted 23 February 1999*

*Correspondence to:* MC Bibby

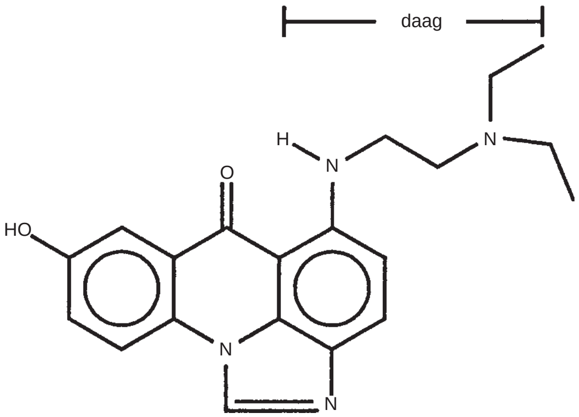

**Figure 1** Structure of the imidazoacridinone C1311, daag = diaminoalkyl group

### MATERIALS AND METHODS

### Materials/Drugs

The 5-(ω-aminoalkyl)amino-8-hydroxyimidazoacridinone C1311 (Figure 1) was synthesized at the Technical University of Gdansk, as described elsewhere (Cholody et al, 1990, 1992). Stock solutions were prepared in dimethyl sulphoxide (DMSO) or normal saline. Aqueous solutions were prepared in polystyrene tubes to minimize adsorption effects and quantified by spectrophotometry. Hoechst 33258 dye (Sigma-Aldrich Ltd) was used as a control ligand for viscometric studies and quantified using $\varepsilon_{338}$ = 42 000 $M^{-1}$ $cm^{-1}$ (Loontiens et al, 1990). All DNA binding experiments were performed in aqueous phosphate buffer (10 mM $NaH_2PO_4/Na_2HPO_4$, 1 mM $Na_2EDTA$, pH 7.00 ± 0.01). Double-stranded calf thymus DNA (CT-DNA, type-I [Sigma-Aldrich Ltd.]) was sonicated and purified using a published procedure (Chaires et al, 1982), then dialysed for 48 h against phosphate buffer using a MW 10 000 cut-off membrane to remove short fragments. This procedure results in duplex DNA with an average length of ~200 bp (~100 kDa) necessary for viscometric studies to ensure that the DNA behaves like a stiff rod and to minimize effects from changes in persistence length (Chaires et al, 1982). CT-DNA solutions were quantified spectrophotometrically using $\varepsilon_{260}$ = 12 824 $M(bp)^{-1}$ $cm^{-1}$.

Lyso tracker™ and Mito tracker™ were obtained from Molecular Probes Europe (Leiden, The Netherlands). Rhodamine 123 dyes and all other chemicals or reagents used were purchased from Sigma-Aldrich Ltd (Poole, Dorset, UK). Tissue culture medium and supplements were purchased from Life Technologies (Paisley, UK) and Costar Ltd (High Wycombe, UK).

### Cell culture and in vitro cytotoxicity assay

The human colon cancer cell line HT29 was obtained from the central repository of the National Cancer Institute in vitro cell line screening programme (Frederick, MD, USA) and was routinely maintained as monolayer culture in RPMI-1640 medium supplemented with 10% fetal calf serum and 2 mM L-glutamine in a humidified atmosphere containing 5% carbon dioxide at 37°C. C1311 doses required to produce 50% ($IC_{50}$) or total growth inhibition (TGI), and the effects of drug concentration (C) and time (T) on chemosensitivity, were determined using a 3-(4,5-dimethylthiazol-2-yl)-2,5-diphenyltetralium bromide (MTT) assay procedure as described elsewhere (Burger et al, 1996; Nicholson et al, 1997).

### Measurement of DNA/C1311 complexes in HT29 cells

Exponentially growing HT29 cells were trypsinized and seeded into 96-well plates (50 000 per well) 24 h prior to drug treatment. Cells were then exposed to C1311 at $IC_{50}$ (0.5 μM) and TGI (2 μM) concentrations for 1, 4 and 8 h. Control cells were treated with vehicle (saline) only. To measure complexes formed by C1311 with genomic DNA, plates were washed twice with 200 μl phosphate-buffered saline (PBS), and C1311 in DNA quantified by using a fluorescence plate reader (Cytofluor 2300, Perkin-Elmer). Fluorescence intensity of C1311-treated HT29 cells (in 100 μl PBS per well) was determined at 360/40 nm excitation and 530/25 nm emission filter settings.

### Fluorescence microscopy studies

HT29 cells were grown on glass slides to follow the intracellular distribution of C1311 at various time points: 30 min, 1 h, 4 h, 8 h, 16 h and 24 h. Exponentially growing cells were treated with 0.5 μM (approx. $IC_{50}$) or 2.0 μM (approx. TGI) drug respectively. Localization of inherent C1311 fluorescence (excitation/emission maxima = 340/520 nm) was compared to the lysosomal paint, Lyso tracker™ (final concentration 200 nM), and the mitochondrial stains rhodamine 123 (10 μg $ml^{-1}$) and Mito tracker™ (final concentration 200 nM) after short-term (30 min) exposure. In addition, C1311 was co-cultured with each of these dyes. Cells were washed three times with ice-cold PBS to remove background for microscopic examination and kept at 4°C. Intracellular fluorescence was documented in a series of fluorescence photomicrographs for each sample and time point using a Leitz DMR binocular fluorescence microscope fitted with a Leica MPS 48/52 photoautomat attachment (Zeiss, Germany).

### Rhodamine 123 release

Rhodamine 123 release as an indicator of mitochondrial impairment and early onset of apoptosis (Yang et al, 1997) was examined using a fluorescence-based 96-well plate assay, following a procedure described previously (Burger et al, 1995). Effects were measured up to 8 h of exposure to $IC_{50}$ and TGI concentrations of C1311.

### DNA binding – thermal denaturation studies

C1311 was subjected to DNA melting studies with calf thymus DNA at a fixed concentration of 50 μM(bp) by adaptation of a reported procedure (McConnaughie and Jenkins, 1995). Working solutions containing CT-DNA and the test compound (2–50 μM) were prepared by addition of concentrated drug solutions in DMSO, to give defined [drug]/[DNA(bp)] molar ratios in the 0.04–1.00:1 range. Samples were monitored at 260 nm using a Varian-Cary 1E spectrophotometer fitted with a Peltier heating accessory. Heating was applied at 1°C $min^{-1}$ in the 5–98°C range. Optical data were imported into the Origin 5.0 computer package (MicroCal Inc., Northampton, MA, USA) for analysis, and DNA

 © 1999 Cancer Research Campaign helix→coil transition temperatures ($T_m$) were obtained from the maxima in the d($A_{260}$)/d$T$ derivative plots. Results are given as the mean ± s.d. from three determinations and are corrected for the effects of DMSO cosolute using a linear correction term (McConnaughie and Jenkins, 1995). Drug-induced alterations in DNA melting behaviour are given by: Æ$T_m$ = $T_m$(DNA + drug) – $T_m$(DNA alone), where the $T_m$ value for the drug-free CT-DNA is 67.8 ± 0.1°C. The [drug]/[DNA] ratios used did not result in binding saturation of the host DNA duplex.

### DNA binding – optical titration experiments

Equilibrium binding constants were determined by spectrophotometric titration of a solution of C1311 (100 μM) with serial aliquots (0.5–5 μl) of concentrated CT-DNA (~8.8 mM in bp) at 25.0 ± 0.1°C using a published procedure (Jenkins, 1997). After each addition and equilibration for 5 min, the absorption spectrum (700–220 nm) was recorded using a Varian-Cary 1E instrument. Addition of aliquots continued until the decreased visible absorbance at 425 nm for the free drug became constant, at which point the addition of DNA itself causes a slight increase. The optical characteristics for binding are as follows: $\lambda_{max}$(free/bound) = 425/430 nm, isosbestic points at 314/335/476 nm. The optical changes at the $\lambda_{max}$ values were analysed to determine the equilibrium distribution of free and bound drug molecules at each data point. The intrinsic DNA–drug equilibrium binding constant $K_i$ was calculated using the following relationship (McGhee and von Hippel, 1974):

$$\frac{r}{C_f} = K_i(1 - nr)\left[\frac{1 - nr}{1 - (n - 1)r}\right]^{n-1}$$

where $r$ is the mole ratio of bound drug to total DNA sites, $C_f$ is the concentration of free ligand and $n$ is the neighbour exclusion parameter or binding site size (Chaires et al, 1982; Jenkins, 1997). Values for $K_i$ and $n$ were determined from binding isotherms using a non-linear fitting routine with the Kaleida Graph 3.08 program (Synergy Software, Reading, PA, USA).

### DNA binding – viscometric studies

Viscosity experiments were carried out in an Ostwald-type capillary viscometer maintained at 25.0 ± 0.1°C by immersion in a circulating water bath (Pilch et al, 1993; Suh and Chaires, 1995). Aliquots of concentrated drug solution were added directly to the viscometer containing a CT-DNA solution [1.3 ml of 250 μM(bp)] in phosphate buffer to give [*total* drug]/[DNA(bp)] molar ratios of 0.075, 0.15, 0.225 and 0.30. These values were selected on the basis of the binding isotherms obtained from the optical titration experiments. Control experiments were performed with Hoechst 33258 dye, an established DNA minor groove-binding ligand (Loontiens et al, 1990; Quintana et al, 1991; Haq et al, 1997).

After mixing by bubbling with a $N_2$ gas stream and thermal equilibration for 30 min, flow times were measured in triplicate to an accuracy of ± 0.1 s; averaged data were used for analysis. For the viscometer used, flow times in the range of 160–220 s were measured after each addition of C1311 ligand. Relative viscosity values were calculated from the observed flow times ($t$) of DNA-containing solutions corrected for the flow time of the buffer alone ($t_0$), using the relation $\eta = (t - t_0)/t_0$. Data are presented as $(\eta/\eta_0)^{1/3}$ versus the experimental binding ratio, where $\eta_0$ is the viscosity of the drug-free DNA solution. This factor approximates to the ratio of relative DNA contour lengths, $L/L_0$, where $L$ and $L_0$ represent the lengths of the rod-like duplex molecule in the presence and absence of added drug.

**Table 1** Effects of drug concentration (C) and time (T) on chemosensitivity in HT29 cells

| Exposure time (h) | C×T value required to kill 50% of cells (μM h⁻¹) ± s.e.m. |
|---|---|
| 1 | 5.0 ± 0.1 |
| 4 | 3.9 ± 0.05 |
| 24 | 0.39 ± 0.01 |
| 144 | 0.36 ± 0.08 |

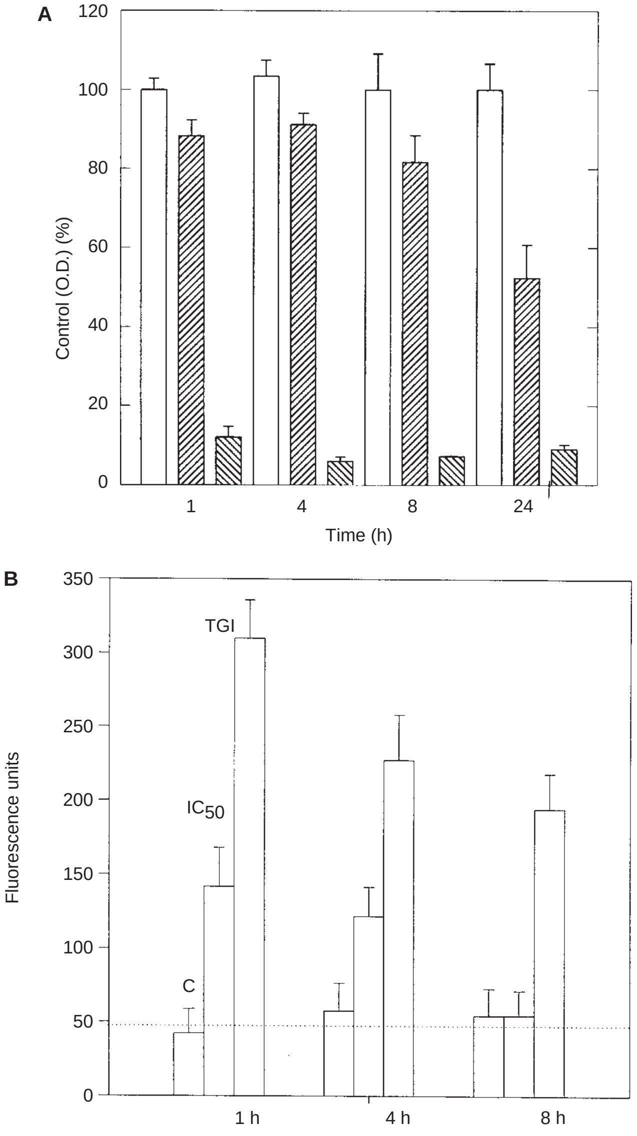

**Figure 2** (**A**) Reversibility of drug effect determined at $IC_{50}$ (▨) and TGI (▧) concentrations of C1311 as compared to vehicle-treated control cells (□), 100% O.D. = 1.25 ± 0.05 (**B**). C1311/DNA complex formation after 1 h, 4 h and 8 h exposure to C1311 at $IC_{50}$ (0.5 μM) and TGI (2 μM) concentration in comparison to vehicle-treated control (**C**) cells. Dotted line indicates non-specific background

### Cytosolic preparations and determination of acid phosphatase activity

Ten million exponentially growing HT29 cells, either untreated (control) or treated with C1311 at concentrations of 0.5 μM and 2.0 μM for 2, 8 or 24 h, were washed twice with ice-cold PBS and homogenized in 0.5 ml of PBS using Kontes tubes. The cell homogenates were spun in a Beckman Optima TL ultracentrifuge at 600 ***g***/4°C for 10 min and then, after removal of the supernatant, at 15 000 ***g***/4°C for 15 min. The supernatant was separately centrifuged at 105 000 ***g***/4°C for 1 h to obtain a cytosolic preparation/cell sap.

Acid phosphatase activity was determined in HT29 cytosolic preparations by using the Sigma Diagnostics Phosphatases kit according to the supplied instruction. In brief: 4-nitrophenyl phosphate in citrate buffer (pH 4.8) was used as substrate and prewarmed to 37°C, then 0.2 ml of cytosol or water (negative control) was added and the mixture incubated at 37°C for 30 min. The reaction was stopped with 5 ml 0.1 M sodium hydroxide and the absorbance was measured at 420 nm using a Beckman DU 650 spectrophotometer. Acid phosphatase units for any individual sample were determined from a calibration curve.

### Apoptosis assay

Exponentially growing HT29 cells on cover slips were treated with 2 μM C1311 for 2, 4, 8 and 24 h. Untreated cells were used as negative control for each time point and another set treated with 40 μM for 48 h to ensure cell death. After each treatment interval, slides were washed with PBS, air dried and cells fixed with 4% formaldehyde in PBS for 30 min. After washing with PBS, cells were subjected to a TUNEL assay using an ApopTag® (S7101 kit, Appligene Oncor, Watford, UK), according to the manufacturer's instructions. Slides were prepared and examined microscopically and the extent of apoptosis was determined from the appearance of peroxidase-labelled apoptotic bodies.

### DNA fragmentation

Exponentially growing HT29 colon cancer cells were cultured in the presence of either 2 μM C1311 (TGI) for 2, 8 and 24 h or 50 μM etoposide (positive control) for 8 h respectively. DNA from $5 \times 10^6$ cells was extracted and analysed following an established protocol (Beckwith et al, 1993). All experiments were performed at least in triplicate.

### RESULTS

### In vitro cytotoxicity and intracellular distribution of C1311

Since C1311 had demonstrated potent in vivo anti-tumour activity in the HT29 human colon cancer xenograft model (Burger et al, 1996), we used this cell line to investigate the mechanism(s) of C1311 antiproliferative activity in intact cells. Cell kinetics studies were performed to determine whether there is a concentration–time dependency (C×T) for the cytotoxic effects of C1311 at $IC_{50}$ and TGI concentrations. The response of HT29 cells to different schedules of C1311 showed no notable differences in cytotoxicity between a 1 h and 4 h exposure period, with C×T values to achieve 50% cell kill of 5.0 and 3.9 μM $h^{-1}$ respectively. However, treatment with C1311 for 24 h was approximately ten times more effective such that the C×T parameter was closely similar to the 6-day value for continuous exposure (Table 1).

In contrast, drug concentrations inducing total growth inhibition (TGI) in a 6-day proliferation assay were already deleterious to HT29 cells after only 1 h of incubation (Figure 2A). This effect on tumour cell growth was paralleled by formation of measurable DNA/C1311 complexes in HT29 cells which reached a maximum at 1-h exposure to TGI doses (Figure 2B). The short period (up to 8 h) of drug reversibility at $IC_{50}$ and the rapid manifestation of cytotoxic lesions and measurable DNA/C1311 complexes at the TGI concentration of C1311 indicate the involvement of a DNA-interactive mode of action for this drug.

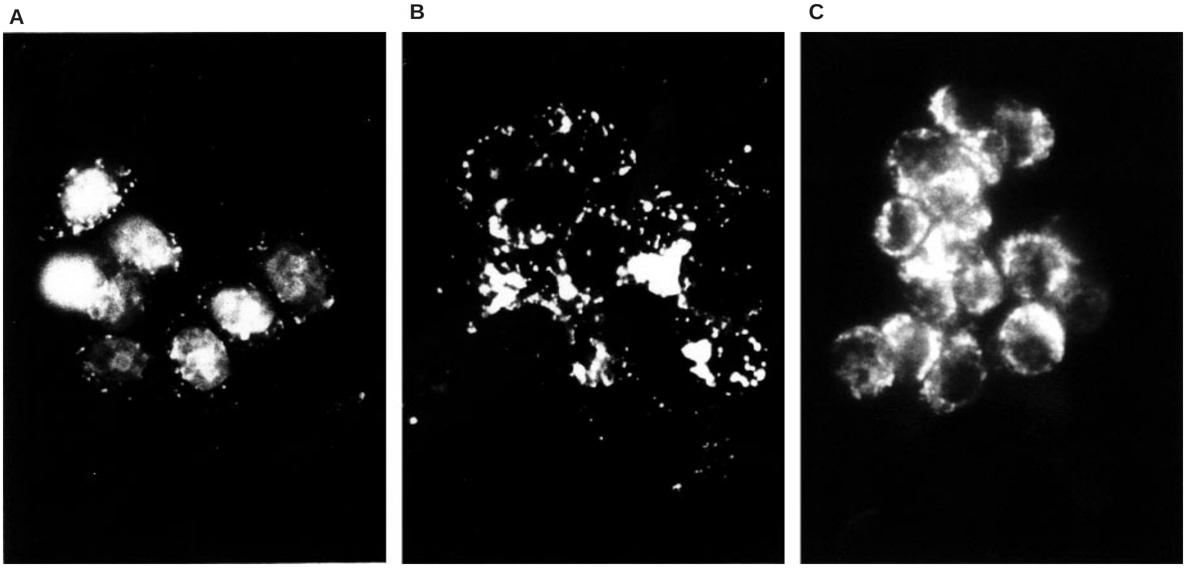

**Figure 3** (**A**) C1311 uptake in HT29 colon cancer cells. (**B**) Labelling of HT29 cells with the lysosomal marker dye Lyso Tracker™. (**C**) Staining pattern in HT29 cells with the mitochondrial membrane probe rhodamine 123. Comparison is shown for dye localization after 30 min exposure to C1311. Magnification 570 ×

 © 1999 Cancer Research Campaign

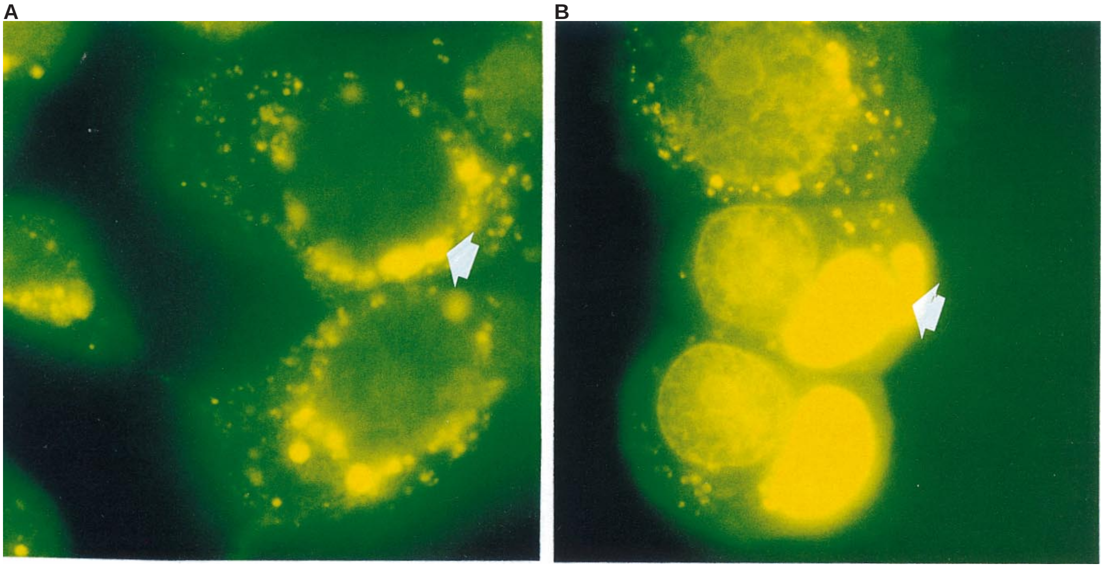

**Figure 4** C1311 accumulation in the lysosomes of HT29 cells. (**A**) Enlargement of lysosomes (white arrowhead) 8 h after exposure to the TGI concentration (2 µM). (**B**) Most of the drug has migrated into the lysosomes and rupture of lysosomes is apparent (white arrowhead) 24 h after continuous exposure to C1311. Magnification 780 ×

Indeed, by following the cellular distribution of inherent C1311 fluorescence we observed a rapid localization of drug in the nuclei of cancer cells (Figure 3A), as noted previously (Berger et al, 1996; Burger et al, 1996; Skladanowski et al, 1996). However, nuclear fluorescence was also accompanied by punctate accumulation in cytosolic organelles and, although cytoplasmic localization was reported earlier (Berger et al, 1996; Burger et al, 1996), its role in C1311 induced tumour growth inhibition remained unclear. The structural features of C1311 suggested to us that the compound would not only be capable of binding to DNA, but may also be trapped into acidic cell compartments due to its basic nature. Thus, due to their protonatable daag side-chains (Figure 1), imidazo-acridinones are likely to accumulate in the lysosomes that provide the most acidic cellular structures. To test this hypothesis, the localization of lysosomal and mitochondrial marker dyes was compared to C1311. Lyso tracker™ showed the same cellular distribution pattern as the C1311 cytosolic concentration (Figure 3A vs 3B), whilst Mito tracker™ or rhodamine 123 (Figure 3A vs 3C) did not match the picture. By studying rhodamine 123 release under C1311 treatment, no effect on mitochondrial membrane integrity was observed (data not shown). The fact that mitochondria were not stained by C1311 and mitochondrial membrane remained intact for more than 8 h under C1311 exposure suggests that these organelles were not targeted. These observations are important in view of current understanding that mitochondria play a pivotal role in controlling cell death in as much that cytochrome C release from mitochondrial membranes is thought to be the initial stimulus of apoptosis cascades (Yang et al 1997; Green and Reed, 1998).

Moreover, following uptake of C1311 in HT29 cells over a time course of 24 h, drug even appeared to migrate from the nuclear compartment into the lysosomes. Lysosomes were markedly enlarged at 8 h under continuous exposure to C1311 and some diffuse cytoplasmic fluorescence resulting from free drug became evident (Figure 4A). Twenty-four hours after drug addition, rupture of 'mega' lysosomes was observed, with C1311 leaking out of these compartments (Figure 4B).

### C1311-induced lysosomal rupture correlates with increase in acid phosphatase activity

Acid phosphatase activity was measured as marker enzyme to examine C1311 lysosomotropism and the resulting lysosomal rupture described above. Enzyme activity was determined 2, 8 and 24 h after exposure to the drug. No difference between control and C1311-treated HT29 cells was seen at 2 h, and no lysosomal rupture was evident at this time point. At 8 h, when the lysosomotropic effect was very prominent, increased acid phosphatase activity was noted in HT29 cells treated at the TGI concentration with 3.03 ± 0.01 U $ml^{-1}$ as compared to a value of 2.17 ± 0.12 U $ml^{-1}$ for the untreated control cells. A significant change to 5.31 ± 0.09 U $ml^{-1}$ was found for cells after treatment

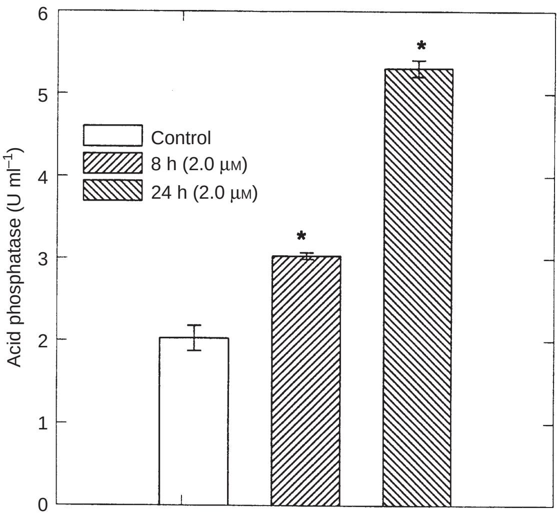

**Figure 5** Acid phosphatase activity in the cytoplasm of HT29 cells untreated (□) or treated with C1311 at TGI concentrations for 8 h (▨) and 24 h (▧) respectively. *$P < 0.05$ (Student's *t*-test)

**Table 2** Binding behaviour of C1311 to calf thymus DNA at pH 7.0

| Æ$T_m$ (°C)[a] at [drug]/[DNA] ratio | | | | | $K_i$[b] | $n$[c] |
|---|---|---|---|---|---|---|
| **0.04:1** | **0.1:1** | **0.2:1** | **0.4:1** | **1:1** | **(×$10^6$ M(bp)$^{-1}$)** | **(bp)** |
| 3.5 | 6.8 | 10.0 | 13.8 | 18.7 | 4.5 ± 0.3 | 3.5 ± 0.1 |

[a]Drug-induced thermal stabilization of CT-DNA (± < 0.2°C) for the [C1311]/[DNA(bp)] molar ratios shown. [b]Intrinsic binding constant determined from optical titration with CT-DNA (see text). [c]Binding site size in DNA base pairs.

with C1311 for 24 h, reflecting the severe lysosomal rupture and collapse of these organelles (Figure 5). However, changes in acid phosphatase activity were not evident for the HT29 cells at $IC_{50}$ concentrations.

### Thermal denaturation studies, binding affinity and stoichiometry

C1311 was assessed as a DNA-binding agent by determination of the thermal helix→coil or melting stabilization (Æ$T_m$) afforded to duplex-form calf thymus DNA. This pseudo-random DNA was selected to overcome possible limitations due to base sequence-dependent binding effects. The compound induced significant stabilization of the DNA duplex relative to the melted strands; Table 2 shows the C1311-induced Æ$T_m$ shifts. Analysis of the differential effects (data not shown) induced upon the G/C or high-temperature (i.e. $T > T_m$) portion of the DNA melting curve relative to the A/T or low-temperature (i.e. $T < T_m$) region suggests possible site- or sequence-preferential binding to AT-rich base segments (McConnaughie and Jenkins, 1995). Factors of ~1.3 are evident from the differential behaviours, particularly at higher [drug]/[DNA] mole ratios where the host duplex is approaching drug saturation. However, such observations can only be qualitative given the thermodynamic limitations of this technique and must await direct observations from sequence foot-printing experiments.

Spectrophotometric titration of calf thymus DNA with C1311 yields an intrinsic binding constant for this interaction of $4.5 \times 10^6$ M(bp)$^{-1}$ with a DNA-binding site size that spans 3.5 base pairs ($r$ = 0.993 for 25 data points). Taken together, these data (Table 2) indicate strongly affinic binding for C1311 to the duplex, with frequent binding sites for this random-sequence DNA.

### Evidence for intercalative DNA binding mode

The criteria for establishing the mode of interaction for DNA ligands have recently been reviewed, indicating that viscosity measurements provide one of the few reliable indicators to distinguish intercalative and groove-mediated binding mechanisms (Suh and Chaires, 1995). Attempted comparative studies of fluorescence quenching and displacement of ethidium bromide were thwarted by the inherent fluorescence of the imidazoacridinone compound C1311. Such indirect fluorometric techniques have previously been used to determine 'apparent' binding constants and qualitative information regarding the binding mechanism for candidate ligands (McConnaughie and Jenkins, 1995; Jenkins, 1997). Thus, instead, we examined the effect of C1311 upon the hydrodynamic properties of DNA.

Figure 6 shows the results from viscometric studies with calf thymus DNA with increasing amounts of either C1311 or Hoechst 33258 for a [drug]:[DNA] range of 0–0.30:1 that corresponds to

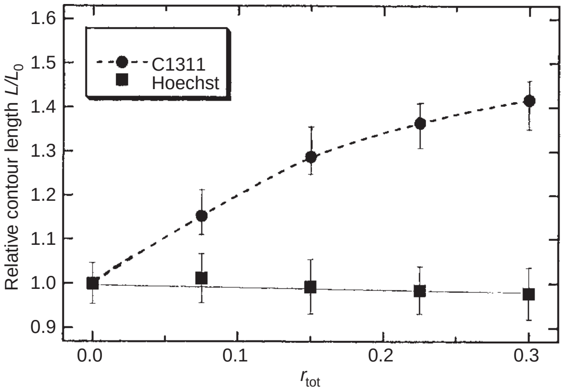

Figure 6 Relative contour length of calf thymus DNA after addition of either C1311 (●) or Hoechst 33258 (■) at the [total drug]/[DNA] ratio shown. The control Hoechst dye, a non-intercalative groove-binding ligand, has little effect whereas C1311 extends the contour length of duplex DNA, as expected for an intercalating ligand non-saturating binding ratios established by titration spectrophotometry. The established minor groove-binding ligand, Hoechst 33258, was used to provide a control for a non-intercalative DNA-binding dye (Loontiens et al, 1990; Vega et al, 1994). For rod-like DNA duplex of length $L$, viscosity is markedly sensitive to length changes (proportional to $L_0$). 'Classical' ligand intercalation would be expected to increase the relative length of the host duplex as has been demonstrated for a number of established intercalating molecules (Suh and Chaires, 1995). Figure 6 shows that C1311 clearly increases the relative contour length of double-stranded CT-DNA, whereas the Hoechst dye effects only a negligible change in the drug dose range examined. These data lead us to conclude that DNA interaction by the imidazoacridinone C1311 involves an intercalative binding mechanism. Further, given the size of the DNA binding site (see above), comparison with established intercalating agents and hybrid combilexin-type ligands suggests that the protonated daag side-chain makes a snug fit into a helical conduit of the DNA duplex (see, for example: Bailly et al, 1989; Agbandje et al, 1992). Such groove accommodation would be expected to achieve favourable electrostatic, hydrophobic and hydrogen-bonded contacts with the host to further stabilize the binding process.

### C1311 and apoptosis in HT29 colon cancer cells

Most anticancer drugs in current use induce apoptosis in susceptible cells (Hickman, 1992). However, it is important to discern the role of apoptosis and the timing of the onset of the cell death cascade in relation to other cellular events. Using the HT29 cell line we could not observe signs of apoptosis by means of impairment of mitochondrial membrane potential (described earlier),

 *© 1999 Cancer Research Campaign*

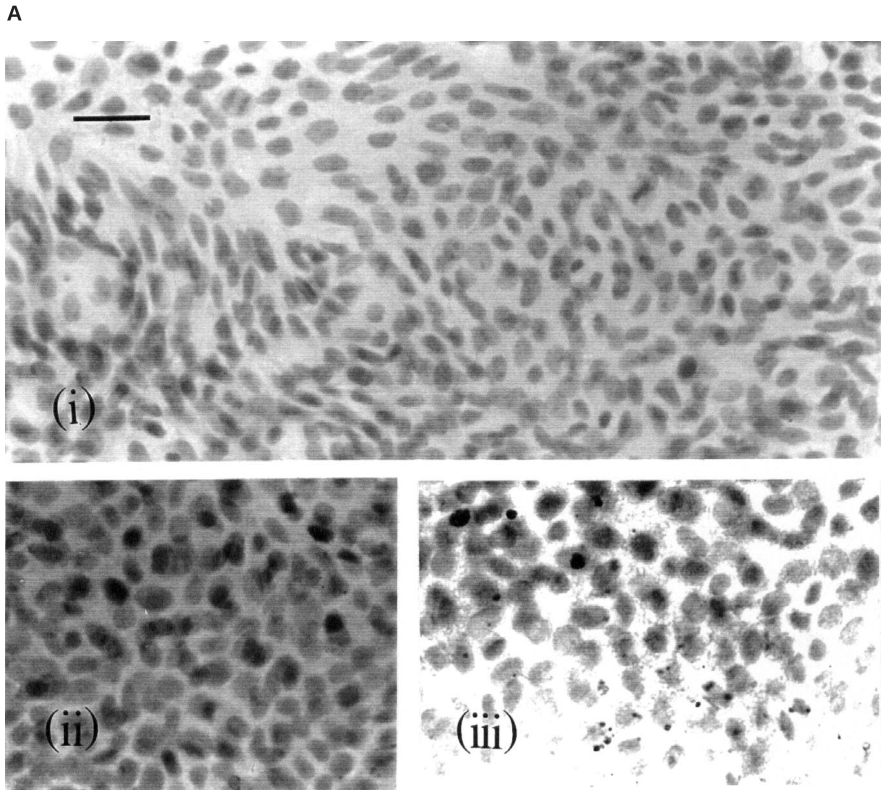

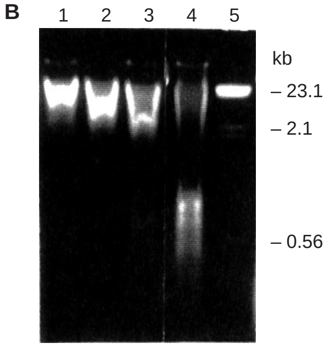

**Figure 7** (**A**) TUNEL (ApopTag) assay for HT29 cells grown on glass slides. Untreated control (i) or treated for 24 h(bar = 20 µM) (ii) with TGI (i.e. 2 µM) C1311 and stained for presence of apoptotic DNA nicks. Positive staining can be seen in the cultures treated with 40 µM C1311 for 48 h (iii). (**B**) Apoptotic DNA ladder. Genomic DNA was isolated from treated cells as described, resolved on a 1.5% agarose gel and stained with ethidium bromide. Lane 1, HT29 cells treated with C1311 at TGI concentration (2 µM) for 2 h; lane 2, for 8 h; lane 3 for 24 h; lane 4, HT29 cells treated with etoposide (50 µM) for 8 h

DNA fragmentation by nick-labelling (ApopTag, Figure 7A) or typifying ladder pattern (Figure 7B) prior to other effects seen such as nuclear uptake of C1311 or lysosomal breakdown. Apoptotic cells were easily identified after treatment with suprelethal C1311 concentrations (40 µM approximately 20X TGI) for 48 h, indicating a successful assay (Figure 7A, iii). We have shown previously that DNA synthesis in HT29 cells is strongly inhibited as early as 1 h after exposure to C1311 at TGI concentrations (Burger et al, 1996), yet the onset of C1311-induced apoptosis in these cells is much delayed and does not occur before 24 h of treatment (Figure 7B). This behaviour indicates that DNA intercalation alone would not cause the potent cytotoxicity seen for C1311. Moreover, as lysosomal swelling is evident at 8 h of continuous exposure to a TGI dose (Figure 4A) and severe rupture is seen after 24 h (Figure 4B), it has to be considered that the apoptosis cascade is not mediated by C1311 directly, but may be triggered indirectly by cell autolysis. In contrast, treatment of HT29 cells with etoposide, an agent known for inducing apoptosis at an early point, resulted in a strong DNA ladder after 8 h of continuous drug exposure (Figure 7B).

### DISCUSSION

C1311 is a new investigational agent that will be evaluated for its potential use in the treatment of colorectal cancer. Thus, to design appropriate trial protocols, it is important to understand how an agent modulates tumour cell growth. Although it is clear from earlier work that DNA and complexes cleavable by topoisomerase II are a cellular target of imidazoacridinone C1311 (Berger et al, 1996; Burger et al, 1996; Skladanowski et al, 1996), the precise mode and strength of DNA binding has not been delineated. Drug accumulation in other than the nuclear compartment, and the cellular kinetics of drug action or the consequences leading to cell death remained largely undefined.

The principal conclusions that can be drawn from this study are as follows: (i) C1311 is a potent inhibitor of HT29 tumour cell

*© 1999 Cancer Research Campaign* 

growth. However, drug effects are schedule- and time-dependent, such that C×T parameters will be an important consideration to achieve clinical efficacy; (ii) C1311 binds to duplex DNA by intercalation, with a high affinity for random-sequence DNA; (iii) the diaminoalkyl structural element also confers a basic nature to C1311 that results in entrapment of the drug in the most acidic cell compartments, the lysosomes (pH 4–5), by an interaction described as acidic shift (Simon et al, 1994). This lysosomotropism of C1311 leads to subsequent lysosomal swelling, rupture and release of autolytic enzymes at concentrations relevant for inhibition of tumour cell growth (Figures 4 and 5); (iv) lysosomal rupture (8 h after treatment at the TGI dose) precedes the onset of apoptosis (not prior to 24 h treatment at TGI) by the means of DNA-fragmentation for more than 16 h (Figure 7). Thus, the lysosomal compartment is broken down and cell autolysis is initiated before apoptosis becomes evident, indicating a central role for induced lysosomal rather than DNA damage in the cascade of C1311-mediated cell death.

Lysosomes and lysosomal enzymes have long been postulated to play a role in cancer. It has been shown that the specific activity of lysosomal enzymes in tumours is elevated as compared to normal tissues. Indeed, shrinkage of neoplasms in response to therapy has been attributed to lysosomes or their products (reviewed in Allison, 1974; Overgaard, 1977; Boyer and Tannock, 1993). In accord, de Duve had proposed in the late 1950s that cells might be killed from within, by an explosion of their lysosomes acting as ‘suicide bags’, and thus that lysosomes may represent a possible target for therapeutic intervention (Majno and Joris, 1995). Although none of the standard anticancer agents currently used in the clinic is thought to kill tumour cells by affecting lysosomes, it has been described that the efficacy of hyperthermia (Overgaard, 1977) and radiation treatment is partly due to lysosomal breakdown and release of lysosomal enzymes, e.g. acid phosphatase, β-glucuronidase and cathepsin B, into the cytoplasm (Boyer and Tannock, 1993). Furthermore, Firestone and co-workers proposed a novel approach to target tumour cells by utilizing lysosomal enzymes and synthesized a new class of agents termed lysosomotropic detergents (Firestone et al, 1979). These compounds are designed to be weakly basic so they concentrate within lysosomes as a result of the pH gradient across the lysosomal membrane. Drug accumulating inside the lysosomes would then dissolve the membranes, releasing lysosomal enzymes into the cytoplasm and result in cell death from autolysis. Furthermore, a similar concept was recently described by Engelke and colleagues (Engelke et al, 1997). These authors observed that 9-aza-anthrapyrazoles with tertiary amine side chains, compounds structurally related to C1311, localized in lysosomes and proposed that they would act by a lysosomal mechanism rather than DNA interaction.

Intercalation alone may not be sufficient for the marked antitumour activity of C1311, and it has been established that the subsequently formed cleavable DNA–protein complexes are reversible and probably not the sole cause of cell death (Skladanowski et al, 1996). Thus, the pronounced cytotoxic effects exerted by C1311 toward tumour cells in vitro and in vivo may in part result from induced lysosomal rupture and the cascade of events leading to cell kill detailed above.

Our kinetic data and fluorescence drug uptake studies, which show cytotoxic effects paralleled by lysosomal drug accumulation and increased lysosomal enzyme activity starting at 8 h of drug exposure, strongly support a lysosomal involvement in the mode of action of C1311.

In conclusion, understanding the role of lysosomes in cancer and targeting these cellular compartments in an anti-cancer strategy by using them as ‘suicide bags’ warrants further investigation.

### ACKNOWLEDGEMENTS

This work was supported in part by the Bradford’s War on Cancer (MCB, JAD), the Cancer Research Campaign and Enzacta Ltd. (TCJ), the ‘Deutsche Forschungsgemeinschaft’ (AMB) and the Yorkshire Cancer Research Campaign (JAD and AMB). MCB and JAD are members of the EORTC-Screening and Pharmacology Group.

### REFERENCES

Agbandje M, Jenkins TC and Neidle S (1992) Anthracene-9,10-diones as potential anticancer agents. Synthesis, DNA-binding, and biological studies on a series of 2,6-disubstituted derivatives. *J Med Chem* **35**: 1418–1429

Allison AC (1974) Lysosomes in cancer cells. *J Clin Pathol* **27**: 43–50

Augustin E, Wheatley DN, Lamb J and Konopa J (1996) Imidazoacridinones arrest cell cycle progression in G2 phase of L1210 cells. *Cancer Chemother Pharmacol* **38**: 39–44

Bailly C, Pommery N, Houssin R and Henichart J-P (1989) Design, synthesis, DNA binding, and biological activity of a series of dna minor groove-binding intercalating drugs. *J Pharm Sci* **78**: 910–917

Beckwith M, Urba WJ and Longo DL (1993) Growth inhibition of human lymphoma cell lines by the marine products, dolastatins 10 and 15. *J Natl Cancer Inst* **85**: 483–488

Berger B, Marquardt H and Westendorf J (1996) Pharmacological and toxicological aspects of new imidazoacridinone antitumor agents. *Cancer Res* **56**: 2094–2104

Boyer MJ and Tannock IF (1993) Lysosomes, lysosomal enzymes and cancer. *Advances in Cancer Research* **60**: 269–291

Burger AM, Kaur G, Alley MC, Supko JG, Malspeis L, Greever MR and Sausville EA (1995) Tyrphostin AG17 [(3,5-Di-*tert*-butyl-4-hydroxybenzylidene)-malononitrile], inhibits cell growth by disrupting mitochondria. *Cancer Res* **55**: 2794–2799

Burger AM, Double JA, Konopa J and Bibby MC (1996) Preclinical evaluation of novel imidazoacridinone derivatives with potent activity against experimental colorectal cancer. *Br J Cancer* **74**: 1369–1374

Chaires JB, Dattagupta N and Crothers DM (1982) Studies on interaction of anthracycline antibiotics and deoxyribonucleic acid: equilibrium binding studies on interaction of daunomycin with deoxyribonucleic acid. *Biochemistry* **21**: 3933–3940

Cheng CC and Cheng RKY (1989) The design, synthesis and development of a new class of potent antineoplastic anthraquinones. In: *Progress in Medicinal Chemistry*, Ellis GP and West GB (eds), pp. 88–113. Elsevier: New York

Cholody WM, Martelli S, Lukowicz J and Konopa J (1990) 5-[(Aminoalkyl)amino]imidazo4,5,1 de]acridin-6-ones as a novel class of antineoplastic agents. Synthesis and biological activity. *J Med Chem* **33**: 49–52

Cholody WM, Martelli S and Konopa J (1992) Chromophore-modified antineoplastic imidazoacridinones. Synthesis and activity against murine leukemias. *J Med Chem* **35**: 378–382

Engelke K, Krapcho P and Hacker M (1997) The intracellular distribution of the 9-aza-anthrapyrazole compounds is regulated by the side-chain terminal amines. *Proc Am Assoc Cancer Res* **38**: 601

Firestone RA, Pisano JM and Bonney RJ (1979) Lysosomotropic agents. 1. Synthesis and cytotoxic action of lysosomotropic detergents. *J Med Chem* **22**: 1130–1133

Green DR and Reed JC (1998) Mitochondria and apoptosis. *Science* **281**: 1309–1312

Haq I, Ladbury JE, Chowdhry BZ, Jenkins TC and Chaires JB (1997) Specific binding of Hoechst 33258 to the $d(CGCAAATTTGCG)_2$ duplex: calorimetric and spectroscopic studies. *J Mol Biol* **271**: 244–257

Hickman JA (1992) Apoptosis induced by anticancer drugs. *Cancer Metastas Rev* **11**: 121–139

Jenkins TC (1997) Optical absorbance and fluorescence techniques for measuring DNA–drug interactions. In: *Methods in Molecular Biology, Vol. 90, Drug–DNA Interaction Protocols*, Fox URF (ed.), pp. 195–218. Humana Press: Totawa, NJ

Konopa J and Skladanowski A (1987) Anthracyclines and anthracenediones induce covalent interstrand DNA crosslinking in tumor cells. In: *Recent Advances Chemotherapy*, Ishigami J (ed.), pp. 663–634. University of Tokyo Press: Tokyo

 © 1999 Cancer Research Campaign

Kusnierczyk H, Cholody WM, Paradziej-Lukowicz J, Radzikowski C and Konopa J (1994) Experimental antitumor activity and toxicity of the selected triazolo- and imidazoacridinones. *Arch Immun Ther Experim* **42**: 415–423

Lamb J and Wheatley DN (1996) Cell killing by the novel imidazoacridinone antineoplastic agent, C-1311, is inhibited at high concentrations coincident with dose-differentiated cell cycle perturbation. *Br J Cancer* **74**: 1359–1368

Loontiens FG, Regenfuss P, Zechel A, Dumortier L and Clegg RM (1990) Binding characteristics of Hoechst 33258 with calf thymus DNA, poly[d(A-T)], and d(CGCGAATTCGCG): multiple stoichiometries and determination of tight binding with a wide spectrum of site affinities. *Biochemistry* **29**: 9029–9039

Majno G and Joris I (1995) Apoptosis, oncosis, and necrosis – an overview of cell death. *Am J Pathol* **146**: 3–15

McConnaughie AW and Jenkins TC (1995) Novel acridine-triazenes as prototype combilexins: synthesis, DNA binding, and biological activity. *J Med Chem* **38**: 3488–3501

McGhee JD and Von Hippel PH (1974) Theoretical aspects of DNA–protein interactions: cooperative and non-cooperative binding of large ligands to a one-dimensional heterogeneous lattice. *J Mol Biol* **86**: 469–489

Nicholson KM, Bibby MC and Phillips RM (1997) Influence of drug exposure parameters on the activity of paclitaxel in multicellular spheroids. *Eur J Cancer* **33**: 1291–1298

Overgaard J (1977) Effect of hyperthermia on malignant cells in vivo. *Cancer* **39**: 2637–2646

Pilch DS, Waring MJ, Sun J-S, Rougee M, Nguyen C-H, Bisagni E, Garestier T and Helene C (1993) Characterization of a triple helix-specific ligand. *J Mol Biol* **232**: 926–946

Quintana JR, Lipanov AA and Dickerson RE (1991) Low-temperature crystallographic analyses of the binding of Hoechst 33258 to the double-helical DNA dodecamer CGCGAATTCGCG. *Biochemistry* **30**: 10294–10306

Simon S, Roy D and Schindler M (1994) Intracellular pH and the control of multidrug resistance. *Proc Natl Acad Sci USA* **91**: 1128–1132

Skladanowski A, Plisov SY, Konopa J and Larsen AK (1996) Inhibition of DNA topoisomerase II by imidazoacridinones, new antineoplastic agents with strong activity against solid tumors. *Mol Pharmacol* **49**: 772–780

Suh D and Chaires JB (1995) Criteria for the mode of binding of DNA binding agents. *Bioorg & Med Chem* **3**: 723–728

Vega MC, Garcia-Saez I, Aymami J, Eritja T, Van der Marel GA, van Boom JH, Rich A and Coll M (1994) Three-dimensional crystal structure of the A-tract DNA dodecamer $d(CGCAAATTTGCG)_2$ complexed with the minor groove-binding drug Hoechst 33258. *Eur J Biochem* **222**: 721–726

Yang J, Liu X, Bhalla K, Kim NC, Ibrado AM, Cai J, Peng TI, Jones DP and Wang X (1997) Prevention of apoptosis by bcl-2: release of cytochrome c from mitochondria blocked. *Science* **275**: 1129–1132

*© 1999 Cancer Research Campaign*
